# Risk factors for shoulder pain after stroke: A clinical study

**DOI:** 10.12669/pjms.38.1.4594

**Published:** 2022

**Authors:** Na Hao, Mingming Zhang, Yuling Li, Yingnan Guo

**Affiliations:** 1 Na Hao, Encephalopathy Department, Hengshui Hospital of Traditional Chinese Medicine, Hengshui City, 053000, China; 2 Mingming Zhang, Encephalopathy Department, Hengshui Hospital of Traditional Chinese Medicine, Hengshui City, 053000, China; 3 Yuling Li, Encephalopathy Department, Hengshui Hospital of Traditional Chinese Medicine, Hengshui City, 053000, China; 4 Yingnan Guo, Encephalopathy Department, Hengshui Hospital of Traditional Chinese Medicine, Hengshui City, 053000, China

**Keywords:** Shoulder pain, Risk factors, Stroke, Clinical study

## Abstract

**Objectives:**

To investigate the risk factors for shoulder pain after stroke, and prevent its occurrence effectively.

**Methods:**

The patients with stroke treated in our hospital between September 2016 and October 2020 were reviewed retrospectively. The medical records of the included patients including age, gender, lesion side, stroke duration, hospital stay, diabetes, hypertension, heart disease, limitation of shoulder joint activity, alcohol abuse, smoking, type of stroke, Ashworth scale, Brunnstrom stage, sensory disorders, and motor arm score of National Institutes of Health Stroke Scale (NIHSS) were collected and analyzed to determine the risk factors for shoulder pain after stroke.

**Results:**

A total of 1390 patients were included based on the inclusion and exclusion criteria, consisting of 162 patients with shoulder pain after stroke and the prevalence was 11.6%. The included patients were divided into shoulder pain group and non-shoulder pain group. There were significant differences in age, stroke duration, hospital stay, diabetes, limitation of shoulder joint activity, Ashworth scale, Brunnstrom stage, sensory disorders, and motor arm score of NIHSS between the two groups (P < 0.05). Based on the multivariate regression analysis, the independent risk factors for shoulder pain after stroke included diabetes, limited shoulder joint activity, Brunnstrom grade I-III period, Ashworth 3-4 grade, motor arm score of NIHSS 3-4 points, and sensory disturbance.

**Conclusion:**

Great emphasis should be placed on the stroke patients with diabetes, limited shoulder joint activity, Brunnstrom grade I-III period, Ashworth 3-4 grade, motor arm score of NIHSS 3-4 points, or sensory disturbance, as these patients have higher risks for shoulder pain after stroke.

## INTRODUCTION

Stroke is one of the main causes of mortality and disability in the world, and it was reported that about 15 million people suffer from stroke each year, which impose a heavy burden on social health care system.^1^ Meanwhile, shoulder pain after stroke, a common and disabling complication, with a prevalence of up to 12%-49%,^2^,^3^ usually occurs two to three months after stroke and may result in withdrawal from rehabilitation programs, longer hospital stays, reduced limb function, impairing quality of life of the stroke patients adversely.^4^ As a result, many physicians focus on the analysis of the risk factors for shoulder pain after stroke and try to understand its etiology, and prevent its occurrence.

In a study including a total of 94 patients with unilateral stroke lesion, clinical, radiological and sonographic evaluations were performed and patients with severe arm paralysis and supraspinatus tendon pathology were found to be at high risk of hemiplegic shoulder pain.^5^ In another study of 2585 patients, Wang and colleagues found the risk factors for shoulder pain after stroke were diabetes, recurring stroke, shoulder activity limitation, Brunnstrom I-III stages, Ashworth scale 3-4 stage, motor arm score of NIHSS 3-4 points, and sensory disturbance.^6^ In addition, some other authors also studied the issue, but they gave different conclusions in their clinical studies.

Moreover, some authors have published meta-analysis to clarify the issue. In a meta-analysis, Shahnawaz and colleagues^4^ concluded that the significant predictors of shoulder pain after stroke were age, female gender, increased tone, sensory impairment, left-sided hemiparesis, hemorrhagic stroke, hemispatial neglect, and poor NIHSS score. However, in another meta-analysis by Holmes^7^, diabetes, a history of shoulder pain, and reduced motor function in the upper limb were identified as significant risk factors for the development of shoulder pain after stroke. Obviously, a wide variation is available in meta-analysis studies. At the same time, both meta-analyses have referred to their limitations, in terms of outcome measures, cause and definition of shoulder pain, and selective reporting^4^,^7^, which may affect the validation of the conclusions inevitably. Up to now, the issue seems still controversial. This condition may affect physicians better understand the occurrence of this disease.

Therefore, in this study, we reviewed retrospectively the patients with stroke treated in our hospital between September 2016 and October 2020, and the aim of our study was to investigate the risk factors for shoulder pain after stroke, and try to prevent its occurrence effectively.

## METHODS

In this study, the patients with stroke treated in our hospital between September 2016 and October 2020 were reviewed retrospectively

### Inclusion and Exclusion Criteria:

The study was approved by the institutional review board of our hospital on January 6, 2021. The inclusion criteria were patients over 18 years who were diagnosed with stroke using CT or MRI and clinical examinations, and gave informed consent.^2^ To facilitate the study, those patients with bilateral stroke were excluded. In addition, patients complicated with coma, severe infection, myocardial infarction, severe hepatic or renal insufficiency, recurring stroke, and severe cognitive impairment, and patients with shoulder pain before stroke were excluded.^5^

The medical records of the patients including age, gender, lesion side, stroke duration, hospital stay, diabetes, hypertension, heart disease, limitation of shoulder joint activity, alcohol abuse, smoking, type of stroke, Ashworth scale, Brunnstrom stage, sensory disorders, and motor arm score of National Institutes of Health Stroke Scale (NIHSS) were collected and analyzed.

In this study, the definition of shoulder pain after stroke was that when a patient reported shoulder pain confined to the shoulder of the hemiplegic side in the resting state or during passive range-of-motion exercise.^5^ As shoulder pain after stroke can be caused by injuries to bones, tendons, ligaments, central and peripheral nerves, the etiology is multifactorial. To clarify the exact etiology of each patient with shoulder pain, X-ray and MRI scan were performed for the affected shoulder in all the included patients, and etiological assessment of shoulder pain was conducted by specialists from neurology, rehabilitation, orthopedics, and imaging departments.

During the process of assessment, the diagnosis of shoulder subluxation was determined by comparing the bilateral vertical distances between the inferior acromial point and the central point of the humeral head, measured on the shoulder X-ray.^5^ The diagnosis of shoulder hand syndrome was based on the criteria including (1) patients have pain in shoulder and hand and swelling of shoulder, hand and fingers with or without mobility limitation on the affected side for at least three months, and (2) patients have no history of trauma, infection or peripheral vascular diseases in the recent past.^8^ The diagnosis of frozen shoulder is according to the criteria in which the patients presenting with an insidious onset of pain and stiffness with a clinical reduction in the range of motion and without an underlying radiologic abnormality.^9^ Additionally, the diagnosis of other common shoulder pain after stroke such as rotator cuff injury, tendon inflammation, and spasm of shoulder joint was all determined based on related literature.^6^

The statistical analysis was performed using SPSS 23.0. The measurement data were represented by mean ± standard deviation, which were compared by the two-sample t-test. The enumeration data were represented by percentage, and chi-square test was used for comparison. The risk factors for shoulder pain after stroke were analyzed by binary logistic regression analysis. Meanwhile, stepwise regression method and forward method were used to enter the regression equation, and p < 0.05 was considered statistically significant.

## RESULTS

A total of 1390 patients were included based on the inclusion and exclusion criteria in this study, consisting of 162 patients with shoulder pain after stroke and the prevalence was 11.6%. According to whether shoulder pain was available, the included patients were divided into two groups, i.e., the shoulder pain group and non-shoulder pain group. Baseline data and clinical characteristics of the two groups are shown in Table-I.

**Table I:** Clinical characteristics of the two groups.

Variables	Shoulder pain group (n=162)	Non-shoulder pain group (n=1228)	p value
Age (Year)	65.6±4.8	53.4±3.7	0.032
Gender (Male/Female)	85/77	699/529	0.283
Hospital stay (day)	23.7±5.6	18.7±3.9	0.002
Stroke duration (day)	45.7±7.8	21.6±4.1	0.001
Hypertension (n, %)	102 (62.9%)	708 (57.7%)	0.199
Diabetes mellitus (n, %)	91(56.2%)	548(44.6%)	0.006
Heart disease (n, %)	35(21.6%)	349(28.4%)	0.07
Paralysis of left limbs (n, %)	89 (54.9%)	646 (52.6%)	0.576
Smoking habit (n, %)	87 (53.7%)	698 (56.8%)	0.554
Alcohol abuse (n, %)	65 (40.1%)	528(42.4%)	0.487
Brunnstrom stage I-III (n, %)	118 (72.8%)	345 (28.1%)	<0.001
Ashworth scale (III-IV) (n, %)	107 (66%)	310 (25.2%)	<0.001
Motor arm(3-4 points)	109 (67.3%)	167 (13.6%)	<0.001
Shoulder movement limitation (n, %)	110(67.9%)	135(10.9%)	<0.001
Sensory disturbance (n, %)	91(56.2%)	462(37.6)	<0.001

**Table II:** The independent risk factors for shoulder pain after stroke.

Factors	OR (95% CI)	p values
Diabetes mellitus	1.39 (1.16-2.89)	0.002
Brunnstrom stage (I-III)	10.67 (5.78-19.43)	0.001
Ashworth scale (III-IV)	9.24 (6.34-15.23)	0.001
Motor arm (3-4 points)	31.55(15.49-46.28)	0.002
Sensory disturbance	3.62(1.77-8.69)	0.002
Shoulder activity limitation	15.21 (5.52-22.94)	0.004

In this study, the common etiologies of shoulder pain after stroke include shoulder subluxation (52 cases, 32.1%), rotator cuff injury (73 cases, 45.1%) and tendon inflammation (88 cases, 54.9%), frozen shoulder (77cases, 47.5%), shoulder hand syndrome (38 cases, 23.5%), spasm of shoulder joint (35 cases, 21.6%) and central pain (18 cases, 11.1%). In the current study, 28.5% of patients had single cause, but the remaining patients (71.5%) had multiple causes for shoulder pain.

There were significant differences in age, stroke duration, hospital stay, diabetes, limitation of shoulder joint activity, Ashworth scale, Brunnstrom stage, sensory disorders, and motor arm score of NIHSS between the two groups (P < 0.05), but no significant differences were found in gender, heart disease, hypertension, side of paralysis of limbs, smoking habit, and alcohol abuse (p>0.05) (Fig I and II). In addition, based on the multivariate regression analysis, the independent risk factors for shoulder pain after stroke included diabetes, limited shoulder joint activity, Brunnstrom Grade I-III period, Ashworth scale 3-4 grade, motor arm score of NIHSS 3-4 points, and sensory disturbance.

**Fig.1:**
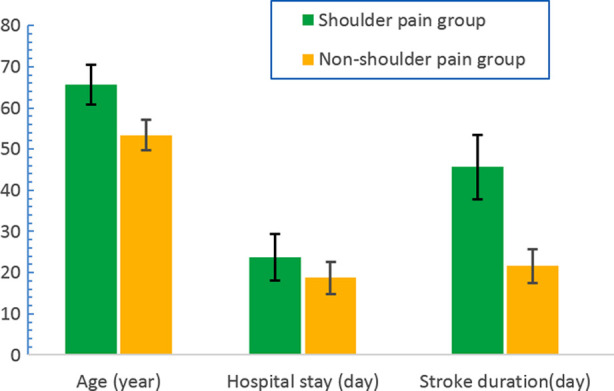
The comparison between the two groups in age, hospital stay and stroke duration.

**Fig.2:**
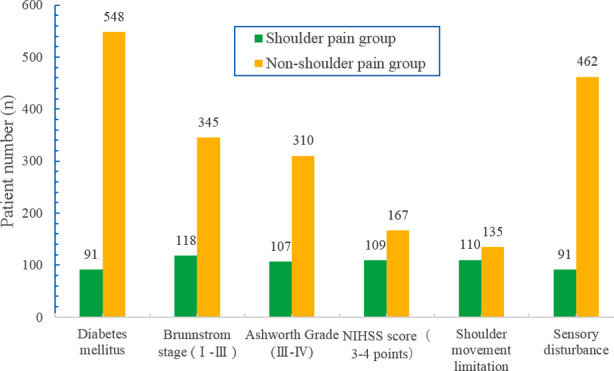
The distribution of the patients in diabetes, Brunnstrom stage, Ashworth grade, NIHSS score, shoulder movement limitation, and sensory disturbance.

## DISCUSSION

In this retrospective study, we investigated the risk factors for shoulder pain after stroke, the study may help physicians understand the mechanism of shoulder pain after stroke and prevent its occurrence correctly.

In terms of the pathomechanism of shoulder pain after stroke, there are different viewpoints available. Some scholars advocated that the primary reason was low tone of muscles, suggesting that during the acute or sub-acute phase of stroke, flaccid paresis of limbs results in potential shoulder disorder, or imbalance of soft-tissue structures, and aggravates altered mechanics and increased susceptibility to injury in shoulder joint.^10^ Different form this viewpoint, some believed that the major reason leading to this type of pain was muscle spasticity, for an instance, Van suggested that during the recovery from stroke, muscle spasticity of the upper extremities is thought to cause shoulder subluxation and limited ROM, resulting in the development of shoulder pain.^11^ In the current study, the two factors including Brunnstrom grade I-III period and Ashworth 3- 4 grade represent relatively higher tone and muscle spasticity, as a result, our results may further confirm the viewpoints of Van.

In addition, we found that the independent risk factors for shoulder pain after stroke included diabetes, limited shoulder joint activity, Brunnstrom grade I-III period, Ashworth scale 3- 4 grade, motor arm score of NIHSS 3-4 points, and sensory disturbance. Our conclusion was partly consistent with Shahnawaz’ study.^4^ However, compared with this study, female gender, age, and left-sided hemiparesis were not found to be independent risk factors in the current study. At the beginning of our analysis, we thought that the outcomes may be attributed to the small sample size of the current study, and the above parameters were evaluated using Chi square test instead of t test or Variance analysis, which may affect the statistical and final results. However, in another meta-analysis article by Holmes,^7^ the parameters including gender, age, laterality, and hemispatial neglect were not found to be significantly risk factors for shoulder pain after stroke, demonstrating that the risk factors may be influenced by multiple factors, instead of sample size alone, even in a meta-analysis study.

Moreover, in this study, we further confirmed the close relationship between diabetes and shoulder pain after stroke, and found diabetes was a significant risk factor. Some published studies found the same facts in patients without stroke. In Alabdali’ s study, a total of 66 patients with type 2 diabetes were included, in which 40.9% of patients were found to have bilateral complaints of symptomatic shoulders, demonstrating a higher prevalence of shoulder pain in diabetes patients.^12^ Kidwai’ study concluded a similar conclusion.^13^ Although the exact mechanism for the increased prevalence of shoulder disorders in diabetes patients remains unclear, some authors advocated that connective tissue damage based on the advanced glycosylation end-products, and peripheral or autonomic neuropathy may play an important role in the occurrence of shoulder pain in patients with diabetes.^12^ In addition, Alabdali found in their study that subacromial pain syndrome was the most prevalent shoulder disorder. Similarly, in the shoulder pain group of the current study, there were 73 cases suffering from rotator cuff injury, 88 cases suffering from tendon inflammation, 77 cases frozen shoulder, and 38 cases shoulder hand syndrome, most of these diseases also belong to subacromial pain syndrome, indicating these two studies demonstrate a similar trend. Subsequently, in this study, the inherent influence of diabetes on shoulder pain combined with muscle spasticity after stroke promote diabetes to be an independent risk factor for shoulder pain after stroke.

### Limitations of the study:

First, different stage of stroke has different characteristics, which may lead to different types of shoulder pain, but in this study, we didn’t evaluate the influence of stoke stage on the issue. Second, multiple factors may affect the occurrence of shoulder pain after stroke, some viewpoints were confirmed in this study, but there are still some different viewpoints available, such as the influence of hemispatial neglect on the occurrence of shoulder pain after stroke, which were not studied for the reason of data collection in this study. Hence, more studies need to be performed in the future.

## CONCLUSION

Great emphasis should be placed on those stroke patients with diabetes, limited shoulder joint activity, Brunnstrom Grade I-III period, Ashworth 3-4 grade, motor arm score of NIHSS 3-4 points, or sensory disturbance, as these patients have higher risks for shoulder pain after stroke.
